# Retraction: Dynamics of droplet motion over hydrophobic surfaces with functionalized and non-functionalized ferro particles

**DOI:** 10.1039/d6ra90017h

**Published:** 2026-02-09

**Authors:** Ghassan Hassan, Bekir Sami Yilbas, Abba Abdulhamid Abubakar, Hussain Al-Qahtani, Abdullah Al-Sharafi

**Affiliations:** a Mechanical Engineering Department, King Fahd University of Petroleum and Minerals (KFUPM) Dhahran 31261 Saudi Arabia bsyilbas@kfupm.edu.sa; b IRC for Renewable Energy and Power, King Fahd University of Petroleum and Minerals (KFUPM) Dhahran 31261 Saudi Arabia; c K. A. CARE Energy Research & Innovation Center Dhahran 31261 Saudi Arabia; d Turkish Japanese University of Science and Technology Istanbul Turkey; e Interdisciplinary Research Center for Advanced Materials, King Fahd University of Petroleum and Minerals Dhahran 31261 Saudi Arabia

## Abstract

Retraction of “Dynamics of droplet motion over hydrophobic surfaces with functionalized and non-functionalized ferro particles” by Ghassan Hassan *et al.*, *RSC Adv.*, 2023, **13**, 34866–34875, https://doi.org/10.1039/D3RA06073J.

The Royal Society of Chemistry hereby wholly retracts this *RSC Advances* article due to concerns with the reliability of the data.

Several concerns with the data have been identified within a group of articles by the same author group.

The authors have not been able to satisfactorily address these concerns.

Given the significance of these concerns, the Editor has lost confidence that the findings presented in this paper are reliable.

The authors were informed about the retraction of the article. Bekir Sami Yilbas has not agreed with the decision, the other authors have not responded.

Bekir Sami Yilbas states that the authors disagree with the retraction and state that all figures were generated by the authors, and the reuse of one or two surface-characterization images neither constitutes duplicated data being presented as new nor compromises the validity of the findings, since the scientific discussion in each paper is supported by multiple figures.

Signed: Laura Fisher, Executive Editor, *RSC Advances*

Date: 30th January 2026

